# Simple linear ionic polysiloxane showing unexpected nanostructure and mechanical properties

**DOI:** 10.1038/s41598-021-97204-8

**Published:** 2021-09-03

**Authors:** Mitsuo Hara, Yuta Iijima, Shusaku Nagano, Takahiro Seki

**Affiliations:** 1 Department of Molecular and Macromolecular Chemistry, Graduate School of Engineering, Nagoya University, Furo-cho, Chikusa-ku, Nagoya, Aichi 464-8603 Japan; 2 Department of Chemistry, College of Science, Rikkyo University, 3-34-1 Nishi-Ikebukuro, Toshima, Tokyo 171-8501 Japan; 3 Present Address: Tosoh Corporation, 1-8 Kasumi, Yokkaichi, 510-8540 Japan

**Keywords:** Polymers, Rheology, Self-assembly

## Abstract

Polysiloxanes are ubiquitous materials in industry and daily life derived from silicates, an abundant resource. They exhibit various properties, which depend on the main-chain network structure. Linear (1D backbone) polysiloxanes provide amorphous materials. They are recognized as fluid materials in the form of grease or oil with a low glass transition temperature. Herein we report that a simple linear polysiloxane, poly(3-aminopropylmethylsiloxane) hydrochloride, shows an elastic modulus comparable to that of stiff resins such as poly(tetrafluoroethylene). By introducing an ammonium salt at all the units of this polysiloxane, inter- and intramolecular ionic aggregates form, immensely enhancing the elastic modulus. This polysiloxane is highly hygroscopic, and its modulus can be altered reversibly 100 million times between moist and dry atmospheres. In addition, it works as a good adhesive for glass substrates with a shear strength of more than 1 MPa in the dry state. Despite its simple structure with a flexible backbone, this polymer unexpectedly self-assembles to form an ordered lamellar nanostructure in dry conditions. Consequently, this work reveals new functions and possibilities for polysiloxanes materials by densely introducing ionic groups.

## Introduction

Poly(organosiloxane)s (simply polysiloxanes or silicones) exhibit various properties, which depend on the network structure of the Si–O–Si main chain. Consequently, polysiloxanes have a wide range of applications from materials chemistry to chemical industry, food, cosmetics, biotechnology, construction, textiles, electronics, and machinery^1–5^. Polysiloxanes with 2D or 3D main-chain networks are used as rubbers. In contrast, linear (1D main-chain) polysiloxanes are featured as grease or oily materials possessing a very low glass transition temperature, typically below − 100°C^1^. The viscosity of linear polysiloxanes increases as the molecular mass increases. To date, there are no examples of vitrified linear polysiloxanes at room temperature.

We recently introduced an ammonium salt into linear polysiloxanes to construct surfactant-polysiloxane hybrid nanostructures, which show humidity-sensitive phase transitions^6,7^. In the course of our explorations, we found that linear polysiloxanes with an ionic group at all siloxane monomer units is vitrified and exhibits a modulus equivalent to that of a typical resin under certain conditions even though it is not chemically crosslinked. For carbon-backbone polymers, introducing ionic groups into polymers results in the formation of ionic aggregates and changes the mechanical and thermal properties of polymers^8–10^. In polysiloxanes, ionic groups are often attached to tune the material properties for diverse applications^11–16^. Several attempts have been made, and in these cases, the amount of ionic groups introduced is less than half of the total monomer units. There are a few examples of linear polysiloxanes with fully introduced ionic groups in all monomer units for electronically functional materials^17^ and fluid polymer ionic liquids^18^, but no studies have examined the mechanical properties.

**Figure 1:**
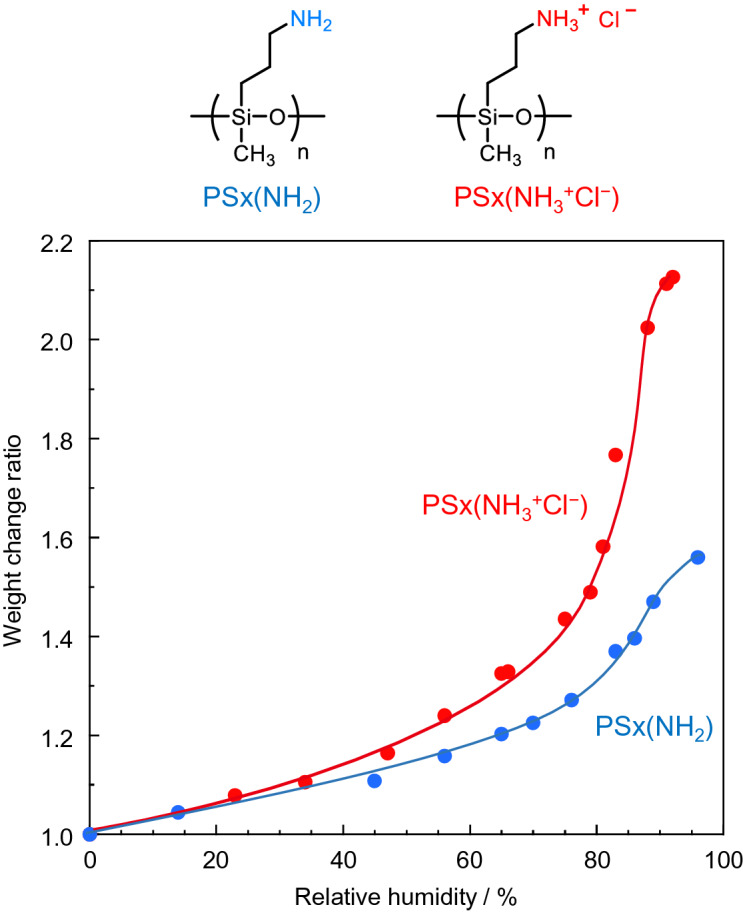
Chemical structures of polysiloxanes and the water vapor uptake amount (weight change ratio) of the polysiloxanes against the relative humidity.

Here, we report for the first time that a simple linear polysiloxane, poly(3-aminopropylmethylsiloxane) hydrochloride (PSx(NH_3_^+^Cl^−^)), displays the modulus of a typical resin (Fig. 1). This polysiloxane material has the following features. First, PSx(NH_3_^+^Cl^−^) shows a 100 million-fold change in the elastic modulus in response to humidity. The elastic modulus, which is initially similar to that of a raw egg, changes to that of a poly(tetrafluoroethylene) (PTFE) resin upon drying. Second, this polysiloxane works as a strong adhesive, exhibiting a shear strength above 1 MPa for bonding glass slides. However, the slides can be readily peeled off after humidification. Third, the polysiloxane possesses an unexpectedly ordered lamellar nanostructure under dry conditions, although the ordering components such as long alkylene chains or mesogens are absent. These unique features should expand toward new utilities of polysiloxane materials, which have been widely used for more than 80 years^1^. Unlike carbon-based polymers made from fossil fuels, polysiloxane materials are made from silicates, an abundant resource^19^. Therefore, the development of polysiloxanes possessing tough mechanical properties should be significant in view of materials sustainability^20,21^.

## Results

### Hygroscopic property

Polysiloxane with ammonium chloride in all monomer units developed in this work (*M*_n_ = 3.2 × 10^3^ and *M*_w_/*M*_n_ = 3.7) is abbreviated as PSx(NH_3_^+^Cl^−^), whereas that of a free amino group is expressed as PSx(NH_2_). The Supplementary Information describes the syntheses of each polymer. The humidity-controlled IR spectrum of PSx(NH_3_^+^Cl^−^) showed an enhanced vibrational absorption of water molecules as the relative humidity increased (Fig. S4). We previously reported that this IR absorption change is not observed in a humidified atmosphere alone^7^. Hence, the increase in the absorption band indicated that the polysiloxanes absorbed water molecules. Figure 1 shows the amount of water molecules absorbed by PSx(NH_3_^+^Cl^−^) and PSx(NH_2_) by changing the relative humidity (RH). The ^1^H NMR spectrum indicate that PSx(NH_3_^+^Cl^−^) and PSx(NH_2_) are fully protonated and deprotonated (Fig. S1).

Although the mass of both PSx(NH_3_^+^Cl^−^) and PSx(NH_2_) increased as RH was enhanced, PSx(NH_3_^+^Cl^−^) exhibited a higher hygroscopicity, especially in the higher RH regions. Since the amount of moisture absorption depends on the hydrophilicity of the hygroscopic groups and the osmotic pressure difference^22,23^, polysiloxane containing a neutralized ionic amine should show a higher moisture absorption than one containing a non-neutralized amine. As a reference, a polymer with a carbon backbone, poly(allylamine) hydrochloride (PAH), was also examined. The moisture uptake by PAH possessing the same hygroscopic group as PSx(NH_3_^+^Cl^−^) absorbed the same amounts of water molecules as PSx(NH_3_^+^Cl^−^) (Fig. S7a).

### Mechanical properties of moist and dry states

**Figure 2:**
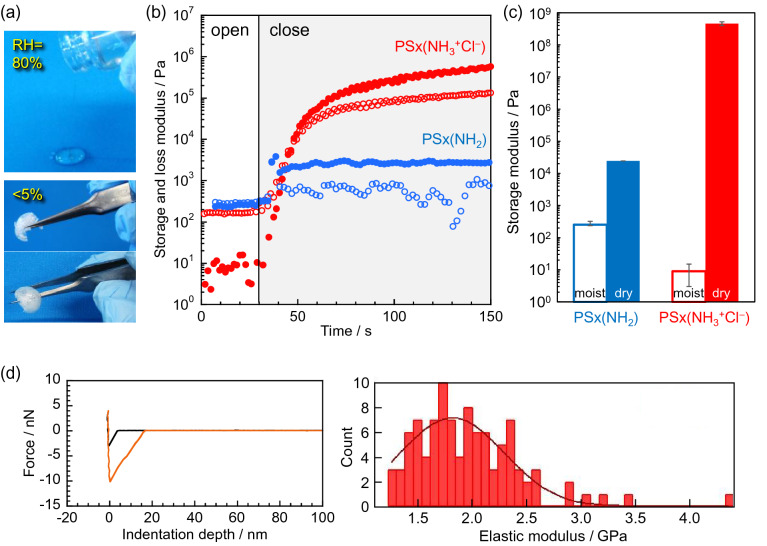
(**a**) Snapshots of the appearance of moist- and dry-PSx(NH_3_^+^Cl^−^). (**b**) Storage and loss moduli changes of PSx(NH_2_) (blue) and PSx(NH_3_^+^Cl^−^) (red) during the drying process. (**c**) Storage modulus of the moist- or dry-state of the polysiloxanes. (**d**) Force curve of dry-PSx(NH_3_^+^Cl^−^) at one point (left) and its Young’s modulus histogram at 100 points (right). Black and orange lines in the left figure show the approach and retraction processes to the film, respectively.

PSx(NH_3_^+^Cl^−^) showed a fluid nature similar to honey in a humid atmosphere at RH = 80%, but in a dry atmosphere (RH < 5%), it became a solid that was rigid enough to be grasped with tweezers (Fig. 2a). The sample equilibrated at RH = 80% was set in a sample chamber under a N_2_ gas flow, and the changes in the storage and loss moduli were recorded during the drying process (Fig. 2b). In Fig. 2b, “closed” in the hatched part indicates the drying process, which began once the chamber was closed. Although drying increased the storage moduli of both PSx(NH_3_^+^Cl^−^) and PSx(NH_2_), the changes for PSx(NH_3_^+^Cl^−^) were more pronounced.

In Fig. 2c, “moist” refers to the storage modulus of the polysiloxanes exposed to RH = 80% environment, while “dry” refers to the modulus at RH < 5%. The absorbed water molecules were completely removed by heating at 200 °C for 30 min. The storage modulus of PSx(NH_2_) modestly increased by 100-fold from the moist state to the dry one. In contrast, the storage modulus of PSx(NH_3_^+^Cl^−^) drastically increased by 100 million times in the dry state.

The higher hygroscopicity is most likely responsible for the lower modulus of PSx(NH_3_^+^Cl^−^) compared to PSx(NH_2_) in the moist state. On the other hand, PSx(NH_3_^+^Cl^−^) showed a higher modulus upon drying, which may be due to the formation of fixed ionic aggregates^24^. Consequently, a large modulus change was observed for PSx(NH_3_^+^Cl^−^) in response to a humidity change. The storage modulus in the moist state was less than that of an egg yolk (< 10^2^ Pa)^25^, although the storage modulus in the dry state was comparable to that of typical synthetic resins such as PTFE (~ 5 × 10^8^ Pa)^26,27^. Linear polysiloxanes exhibiting such a high storage modulus have yet to be reported.

The elastic modulus of PSx(NH_3_^+^Cl^−^) was further evaluated by force curves-distance measurements in the repeated indentation and retraction processes using an atomic force microscopy (AFM) cantilever tip (Fig. 2d). The dry-PSx(NH_3_^+^Cl^−^) showed an elastic modulus of 1.8 × 10^9^ Pa, which agrees well with the storage modulus. This result indicates that the dry-PSx(NH_3_^+^Cl^−^) behaves as a sufficient elastic body. We also confirmed that the modulus of PSx(NH_3_^+^Cl^−^) changes reversibly with the relative humidity (Fig. S5).

### Thermal properties of dry-PSx(NH_2_)

**Figure 3:**
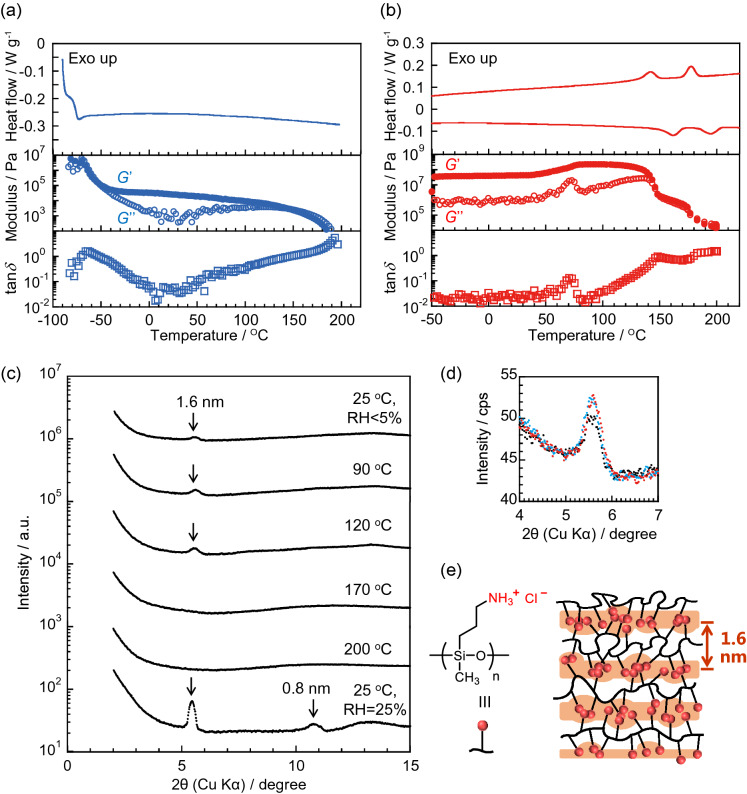
DSC curves and modulus profiles. (**a**) Dry-PSx(NH_2_) and (**b**) dry-PSx(NH_3_^+^Cl^−^). (**c**) Temperature- and humidity-controlled XRD profiles of PSx(NH_3_^+^Cl^−^). (**d**) Magnified and overlay of the XRD profiles in a; black: 25 °C, RH < 5%, red: 90 °C, light blue: 120 °C. (**e**) Schematic of the plausible lamellar structure model of dry-PSx(NH_3_^+^Cl^−^).

Figure 3a shows the results of the thermal analysis of dry-PSx(NH_2_). The baseline shift indicating the glass transition of the polymer was observed around − 80 °C, which is consistent with the data from a molecular dynamic study^28^. Enthalpy changes were not observed up to 200 °C. This is indicative of the amorphous state composed of globular shaped chains estimated by the molecular dynamic study^28^. The storage and loss moduli, which decreased with increasing temperature, showed a long plateau region (–40–150 °C) called the rubbery plateau zone. This behavior is often observed in measurements of physically crosslinked polymers^29^. The long plateau suggests that the weak hydrogen bonds among amine groups in PSx(NH_2_) are maintained up to a high temperature region of about 150 °C.

### Thermal properties and nanostructure of dry-PSx(NH_3_^+^Cl^−^)

The thermal properties of dry-PSx(NH_3_^+^Cl^−^) showed remarkable differences compared with those of dry-PSx(NH_2_) (Fig. 3b). Interestingly, PSx(NH_3_^+^Cl^−^) showed two clear enthalpy changes in the higher temperature region above 140 °C.

Unexpectedly, PSx(NH_3_^+^Cl^−^) formed a well-ordered nanostructure in dry conditions. The temperature-controlled X-ray diffraction (XRD) measurements for PSx(NH_3_^+^Cl^−^) contained a scattering peak corresponding to a *d* spacing of ca. 1.6 nm (2*θ* = 5.4°) at 25 °C and RH < 5% (Fig. 3c). Increasing the relative humidity to 25% improved the nanostructure order, and the first scattering peak became sharper. In addition, a second order peak was observed. On the other hand, the scattering peaks disappeared in the higher humidity regions (Fig. S6c for RH = 80%). With regard to PSx(NH_2_), a peak was not observed under the same conditions (Fig. S6a). These results suggest that PSx(NH_3_^+^Cl^−^) forms an alternating stacked lamellar structure composed of ion aggregation layers and siloxane backbone layers (Fig. 3e).

The improved regularity of the lamellar structure upon mild humidification is probably due to a modestly allowed relaxation of the strong aggregation of ammonium salts by the absorbed water. The PAH (carbon-backbone) did not form a nanostructure in the same water content (Fig. S7b). Therefore, the flexibility of the polysiloxane is attributed to the nanostructure formation. Kaneko et al. reported that a hexagonal structure was observed for an ammonium-containing rigid ladder-structured poly(silsesquioxane)^30^. In our case, a clear nanostructure formed for PSx(NH_3_^+^Cl^−^) possessing the flexible backbone. Thus, the nanostructure is distinctive.

In the dynamic rheological measurements, PSx(NH_3_^+^Cl^−^) showed an absorption, which is often observed as *α*-relaxation around 70 °C in the loss modulus profile^31^. The corresponding storage modulus increased above this temperature (Fig. 3b). The X-ray scattering peak intensity at 90 °C increased slightly (Fig. 3d). This suggests that the lamellar structure is more ordered due to the transition from a frozen state to a more plasticized one at approximately 70 °C, which increases the mobility of the siloxane main chains.

In addition to the endothermic peak in the DSC curve around 150 °C, the storage and loss moduli clearly decreased at 170 °C (Fig. 3b). This behavior is probably due to the melting (disordering) of the lamellar structure. Although the XRD profile indicated no change at temperatures ranging 170–200 °C, an endothermic peak was observed around 180 °C. Ionic aggregates remained even after lamellar structure melting, and the aggregates probably dissociated around 180 °C. In the modulus curves of PSx(NH_3_^+^Cl^−^), the storage and loss moduli decreased in two steps, corresponding to the disorder of the lamellar structure (first relaxation) and the dissociation of the ionic aggregates (second relaxation). It was confirmed by TGA measurement that PSx(NH_3_^+^Cl^−^) does not thermally decompose at 200 °C (see Fig. S9).

The addition of hydrochloric acid into PSx(NH_2_) resulted in identical thermal properties and nanostructures as those of PSx(NH_3_^+^Cl^−^) (Fig. S6b). Thus, the unique properties are attributed to the ionic side structure, and the fluid amorphous PSx(NH_2_) can be converted to the rigid nanostructured PSx(NH_3_^+^Cl^−^) on demand.

### Shear adhesive properties

**Figure 4:**
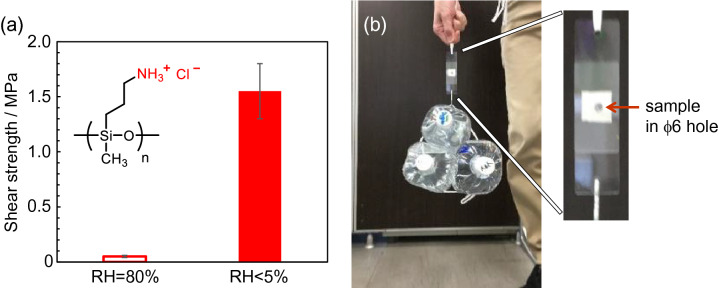
(**a**) Shear strength of moist- and dry-PSx(NH_3_^+^Cl^−^) and (**b**) snapshot of the hanging test for dry-PSx(NH_3_^+^Cl^−^). Arrow in the right enlarged picture indicates the polymer is in the ϕ6 mm circle. This small area fixation can hoist a 6-kg weight.

PSx(NH_3_^+^Cl^−^) was sandwiched between two glass slides for the tensile shear adhesion test. Figure S8 schematically depicts the test piece. PSx(NH_3_^+^Cl^−^) was placed within a 6-mm diameter hole between two glass slides. When PSx(NH_3_^+^Cl^−^) was exposed to the environment of RH = 80%, the shear strength was approximately 0.05 MP (Fig. 4a). The glass slides were readily peeled off by the weight of a ballpoint pen (Supplementary Movie 2). On the other hand, dry-PSx(NH_3_^+^Cl^−^) showed a shear strength of ca. 1.55 MPa (Fig. 4a), and could suspend a 6-kg weight even though the adhesion area was only 28.3 mm^2^ (Fig. 4b, Supplementary Movie 1).

An unexpected strong adhesive property occurred for a linear polysiloxane material. Highly dense multipoint ionic crosslinking probably occurred between the intra and inter side groups of PSx(NH_3_^+^Cl^−^). The glass surface adhesion should be attributed to this marked effect. Changing the humidity reversibly switched the adhesive force. At higher temperatures, the water molecules desorbed from PSx(NH_3_^+^Cl^−^), increasing the adhesive force in a reversible way. This situation is the opposite of general thermal plastic polymers in which the adhesive force decreases at higher temperatures. The observed behavior is similar with those of thermosetting polymers, but thermosets are irreversibly crosslinked by chemical bonds. The unique reversible thermosetting property of PSx(NH_3_^+^Cl^−^) should provide new applications in adhesive technologies.

## Discussion

This study proposes a linear ionic polysiloxane as a new class of silicone materials that exhibits an unexpected self-assembled nanostructure and striking mechanical properties. The mechanical and adhesive properties can be tuned on-demand to an unexpectedly large extent (100-million times) by controlling the atmospheric humidity and temperature. The significance of this study is as follows. First, the polymer is readily synthesized in one pot without special conditions under an ambient atmosphere using industrial silane chemicals. Second, this simple strategy should have great impact because various types of silane compounds are readily available for modifications of solid surface silicone materials in industry. Therefore, diverse polymer designs and functionalization are feasible. Third, the Si–O chemical bond is characterized by its high thermal and chemical stability, inflammability, and non-oxidizable nature. These lead to distinctive features^1–5^ that cannot be obtained using carbon-based polymers.

On the other hand, from scientific viewpoints, the introduction of ionic groups in each Si–O unit of polysiloxane provides an ordered nanostructure. This feature deserves further explorations as a self-assembly chemistry. Currently, studies are underway to elucidate the role of nanostructure formation of PSx(NH_3_^+^Cl^−^) in the emergence of the unique mechanical properties.

## Methods

### Materials

PSx(NH_2_) and PSx(NH_3_^+^Cl^−^) were synthesized from 3-aminopropyldimethoxymethylsilane. The Supplementary Information provides details of the syntheses.

### Water absorption measurements

Approximately 50 mg of polysiloxane was exposed to various humidity-controlled environments for several days. Once it reached an equilibrium moisture absorption state, its weight was promptly measured. The relative humidity was controlled using a saturated aqueous solution of inorganic salts such as lithium chloride, potassium acetate, potassium carbonate, sodium bromide, potassium iodide, sodium chloride, and potassium sulfate^32,33^.

### Dynamic rheological measurements

The dynamic rheological properties were determined by an ARES-G2 Rheometer (TA Instruments) and Discovery HR-2 (TA Instruments) equipped with a ϕ8 mm parallel plate in the oscillation mode. For measurements during the drying process, a polysiloxane sample was placed in a chamber at RH = 80%. The moist polymers were set onto a parallel plate, and the gap was placed at 0.5–1.0 mm. The measurements were performed with 0.5% stain at a frequency of 1 Hz. The chamber door was closed 30 s after the start of the measurement, and N_2_ gas flowed into the chamber to dry the polymer sample. For the temperature-sweep measurements, the moist polymer sample was dried on the parallel plate at 200 °C for 30 min under a N_2_ atmosphere. The gap was set at 0.5–1.0 mm. The samples were cooled from 200 to − 75 °C at a rate of 5 °C min^−1^ with 0.1% strain (for PSx(NH_2_)) or 0.5% strain (for PSx(NH_3_^+^Cl^−^)) at a frequency of 1 Hz.

### Force curve measurements

A spin-cast film (300-nm thick) of PSx(NH_3_^+^Cl^−^) was prepared, and a topographical image was obtained in a 10** × **10 µm^2^ area using a MFP-3D Origin AFM (Asylum Research, Oxford Instruments) under a N_2_ atmosphere (RH < 5%). Force curves obtained by indentation-retraction cycles were measured 10** × **10 (100) times in the image at a lateral scanning speed of nm s^−1^. The cantilever was OMCL-AC240TS-R3 (Olympus; Al-coated silicon, tip radius: 7 nm, force constant: 2 N m^−1^, Frequency: 70 kHz). The spring constant was calibrated in the measurement for a mica surface. By analyzing the retraction process of the cantilever with the JKR model, the elastic modulus was determined.

### Differential scanning calorimetry

For differential scanning calorimetric (DSC) measurements, Q200 (TA Instruments) was used at a 5 °C min^−1^ rate under 50 mL min^−1^ of N_2_ flow. Prior to the measurements, the polysiloxane samples were dried overnight at 100 °C under a vacuum.

### X-ray diffraction measurements

The nanostructure of the polysiloxanes was evaluated by X-ray diffraction (XRD) measurements using FR-E equipped with a two-dimensional detector R-axis IV (Rigaku) involving an imaging plate (Fujifilm). An X-ray beam from Cu Kα radiation (λ = 0.154 nm, 0.3-mm collimated) was used, and the camera length was set at 300 mm. The polysiloxane films were prepared by spin-coating from a methanol solution onto a UV-O_3_ cleaned Si wafer. The polymer concentration of the solution was 5 wt%, and spin casting was performed at 2000 rpm for 30 s. Film samples were placed onto a pulse motor stage and covered with a homemade chamber. The chamber humidity was controlled by a precise dew point generator me-40DP series (Micro Equipment). The incident angle of the X-ray beam was adjusted at 0.18–0.22° to the substrate surface using a Z-pulse motor stage ALV-300-HM and an oblique pulse motor stage ATS-C310-EM (Chuo Precision Industrial). The temperature was controlled by a ceramic heater embedded in the pulse motor stage. The out-of-plane region in the 2D image was used to draw a 2θ-intensity graph.

### Shear adhesive force measurements

Figure S8 depicts the test piece. Two glass slides S111 (Matsunami Glass, 0.9-mm thick) with a ϕ4-mm hole at one end were washed with acetone or a UV-O_3_ cleaner. Then a glass cross tape 361 (3M; 0.19-mm thick) with a ϕ6 hole at the center was attached onto one of them. The depression in the center of the tape on the glass slide was filled with moist PSx(NH_3_^+^Cl^−^) and covered with another glass slide. For shear adhesive force measurements of moist PSx(NH_3_^+^Cl^−^), PSx(NH_3_^+^Cl^−^) was placed in the chamber at RH = 80% a few days in advance. The shear strength was measured by fixing one end of the laminated glass slide and pulling the other end parallel with a digital force gage DST-500N (Imada). To evaluate the shear strength of a target polymer sample, the above procedures were repeated five times. For measurements of dry PSx(NH_3_^+^Cl^−^), after filling the naturally hygroscopic PSx(NH_3_^+^Cl^−^) into the depression and laminating it with another glass slide, a weight (250 g) was placed on it. Then the sample was heated at 100 °C for 3 h under a vacuum to remove the adsorbed water from PSx(NH_3_^+^Cl^−^).

## Supplementary Information

Supplementary Information 1.

Supplementary Video 1.

Supplementary Video 2.
