# Supplementary material for: Simple linear ionic polysiloxane showing unexpected nanostructure and mechanical properties
Source: Sci Rep. 2021 Sep 3;11:17683. doi: 10.1038/s41598-021-97204-8 (PMC8417032; doi:10.1038/s41598-021-97204-8)
Supplement: Supplementary file 1 — Supplementary Information 1. [file 41598_2021_97204_MOESM1_ESM.pdf]

Supplementary Information

Simple linear ionic polysiloxane showing unexpected nanostructure and mechanical properties

Mitsuo Hara*[1], Yuta Iijima[1], Shusaku Nagano[2], and Takahiro Seki*[1]

[1] Department of Molecular and Macromolecular Chemistry, Graduate School of Engineering, Nagoya University, Furo-cho, Chikusa-ku, Nagoya, Aichi 464-8603, Japan

[2] Department of Chemistry, College of Science, Rikkyo University, 3-34-1 Nishi-Ikebukuro, Toshima, Tokyo 171-8501, Japan

Author information

*(M.H.) E-mail: mhara@chembio.nagoya-u.ac.jp

*(T.S.) E-mail: tseki@chembio.nagoya-u.ac.jp

Materials

Triethylamine (TEA) and Sodium nitrate ($NaNO_3$) were purchased from Kishida Chemical. 3-Aminopropyldimethoxymethylsilane (APDMOS) and tris(2,4-pentanedionato) chromium (III) were purchased from Tokyo Chemical Industry. Methanol and concentrated hydrochloric acid (HCl) were purchased from Kanto Chemical. Deuterated methanol (CD_3OD) was purchased from Cambridge Isotope Laboratories. All reagents used were commercial purities. Pure water (PW) was obtained by a Direct-Q® 3 UV purification system (Millipore, ρ >18 MΩ·cm at 25 °C).

Measurements

^{1}H NMR measurements were performed by a 400 MHz FT-NMR spectrometer JNM-A400 (JEOL). CD_3OD containing 30 mmol L^{-1} of tris(2,4-pentanedionato)chromium(III) was used as a solvent. ^{29}Si NMR measurements were performed by a 500 MHz FT-NMR spectrometer Ultra Shield (Bruker). The number of integrations was set to 128, and the relaxation time was set to 10 seconds.

Size exclusion chromatography (SEC) measurements were taken by a HPLC pump Chromaster 5110 (Hitachi) attached with an RI detector Chromaster 5450 (Hitachi). Shodex Protein® KW-802.5 (Showa Denko) was used as LC column and the polymer was characterized by poly(ethylene oxide) standard in mixture solution of 50 mmol L^{-1} $NaNO_3$ aqueous solution and methanol (30:70, v/v) with 1 mL min^{-1}. The LC column was maintained at 30 °C by using an oven CTO-20A (Shimadzu).

Fourier transform infrared (FT-IR) spectroscopy measurements were recorded on a 640-IR (Varian) by changing the humidity. Humidity in the chamber was controlled by a precise dew point generator me-40DP series (Micro Equipment) and monitored by RTR-503 (T&D). For

IR measurements, the polysiloxane films were prepared by spin-coating of a methanol solution (polymer concentration: 5 wt %) onto the UV-O_3 cleaned Si wafers (CZ-grown p-type (100) silicon crystal doped with boron in the order of 5−20 Ω·cm). The spincast condition was set at 800 rpm for 120 s. The film samples were set into CaF_2 chamber. The spectral resolution was set at 4 cm^{-1}.

Thermogravimetric analysis (TGA) was taken by a DTG-60 (Shimadzu). The sample was heated up to 500 °C under 50 mL min^{-1} of N_2 flow, but held at 110 °C for 1 h to avoid adsorbed water. Heating rate was set at 5 °C min^{-1}.

Syntheses of hygroscopic polysiloxanes

PSx(NH_2) was synthesized by sol-gel reaction of APDMOS under basic condition as described below. 1.84g of APDMOS (1.1×10^{-2} mol), 6.86 g of triethylamine (6.8×10^{-2} mol), and 4.58 g of PW (2.5×10^{-1} mol) were placed into a glass bottle, then the mixture was stirred at 70 °C for 5h. After the solvents were evaporated from the mixture, residual viscous liquid was vacuum dried overnight at 40 °C. Final product was 1.5 g of clear viscous liquid.

PSx($NH_3^+Cl^-$) was synthesized by sol-gel reaction of APDMOS under acidic condition. 2.00 g of APDMOS (1.2×10^{-2} mol) and 3.54 g of HCl (3.6×10^{-2} mol) were placed into a glass bottle. This mixture was stirred at r.t. for 2h, successively stirred in open system at 70 °C until all of the solvent evaporated. Residual viscous solid was re-dissolved in a small portion of methanol and vacuum-dried for overnight, then 1.89 g of PSx($NH_3^+Cl^-$) was obtained.

^{1}H NMR spectra and ^{29}Si NMR spectra of the two-types polysiloxanes were shown in Fig. S1 and S2, respectively. In ^{29}Si NMR spectra, only the chemical shifts of a D unit structure of silicone were observed. The NMR spectra show the formation of polysiloxane without branched structure. Molecular weight and its distribution were characterized by SEC measurements. The SEC chart of PSx($NH_3^+Cl^-$) was shown in Fig. S3. Broad peak and overlapping sharp peak were detected. The entire peak was obtained corresponding to M_n = 3.2×10^3 and M_w/M_n = 3.67. The sharp peak is assignable to a ring polymer formation.

X-ray diffraction (XRD) measurements were taken by the same equipment described in the main manuscript.

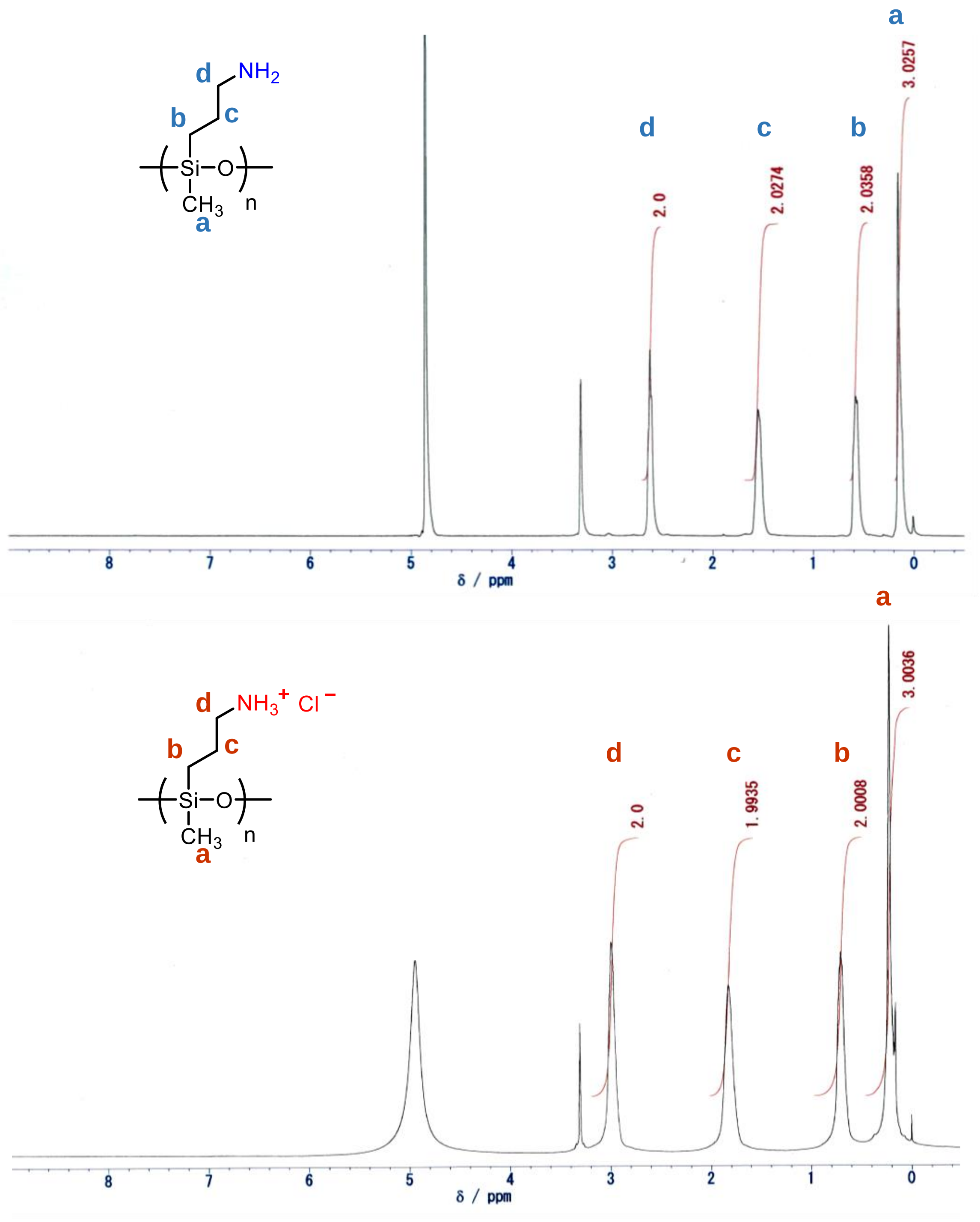

Fig. S1 ^{1}H NMR spectra of PSx(NH_2) and PSx($NH_3^+Cl^-$).

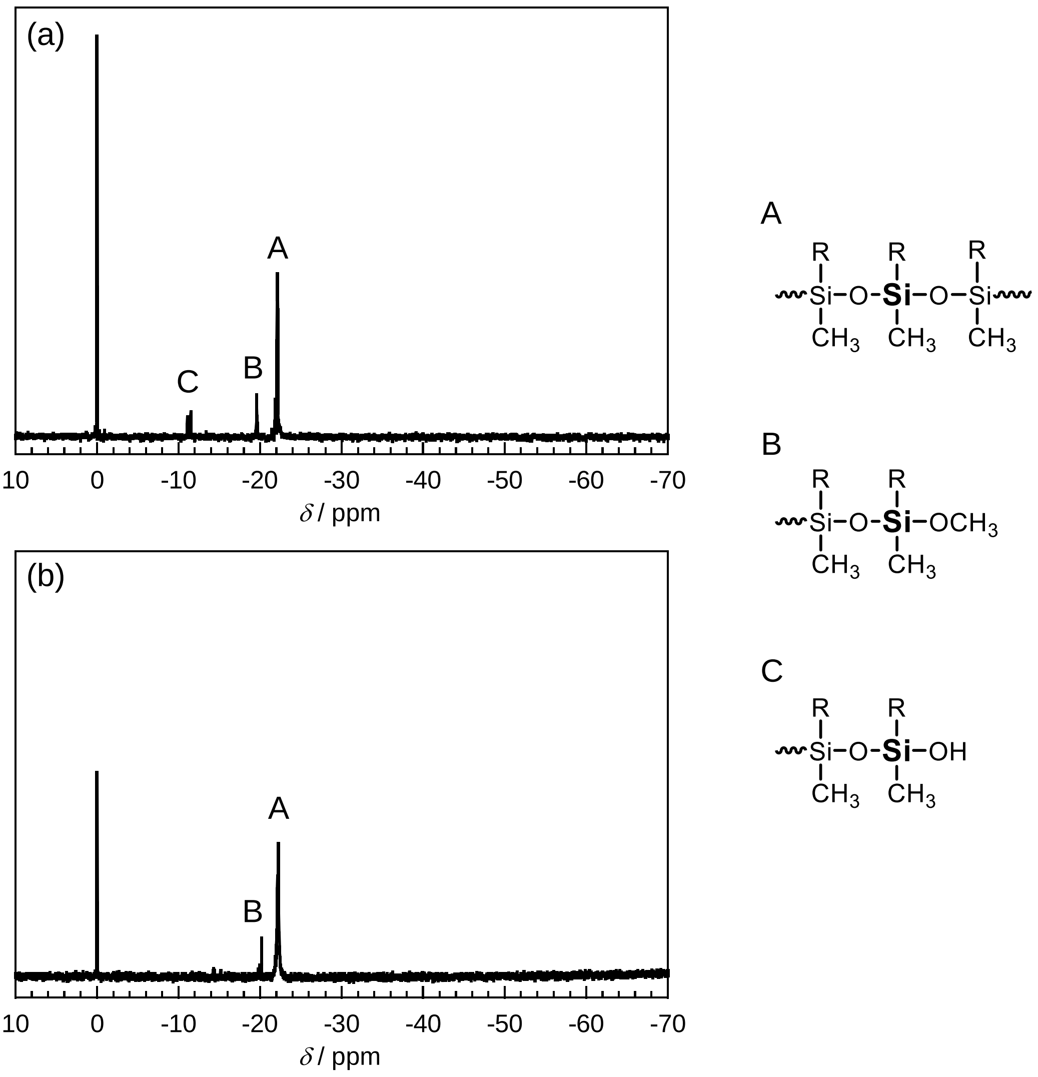

Fig. S2 ^{29}Si NMR spectra of (a) PSx(NH_2) and (b) PSx($NH_3^+Cl^-$).

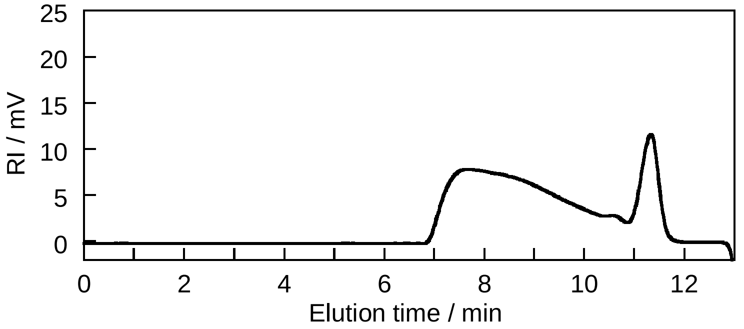

Fig. S3 SEC chart of PSx($NH_3^+Cl^-$).

Hygroscopic nature of the polysiloxane

Humidity-controlled IR spectra of the polysiloxane films on humidification process were shown in Fig. S4. The absorbance of the OH stretching vibration (ν(OH)) around 3410 cm^{-1} and the OH bending vibration (δ(OH) at 1640 cm^{-1} and 2δ(OH) around 3250 cm^{-1})[1,2] increased on humidification process. The water peaks were not observed for the Si wafers only at RH = 90%, indicating that the assigned peaks can be attributed to water taken into the film. The increase in absorbance of PSx($NH_3^+Cl^-$) was larger than that of PSx(NH_2). This is because the increase depends on the amount of moisture absorbed. This is due to the fact that PSx($NH_3^+Cl^-$) absorbs more moisture than PSx(NH_2), as shown in Fig. 1.

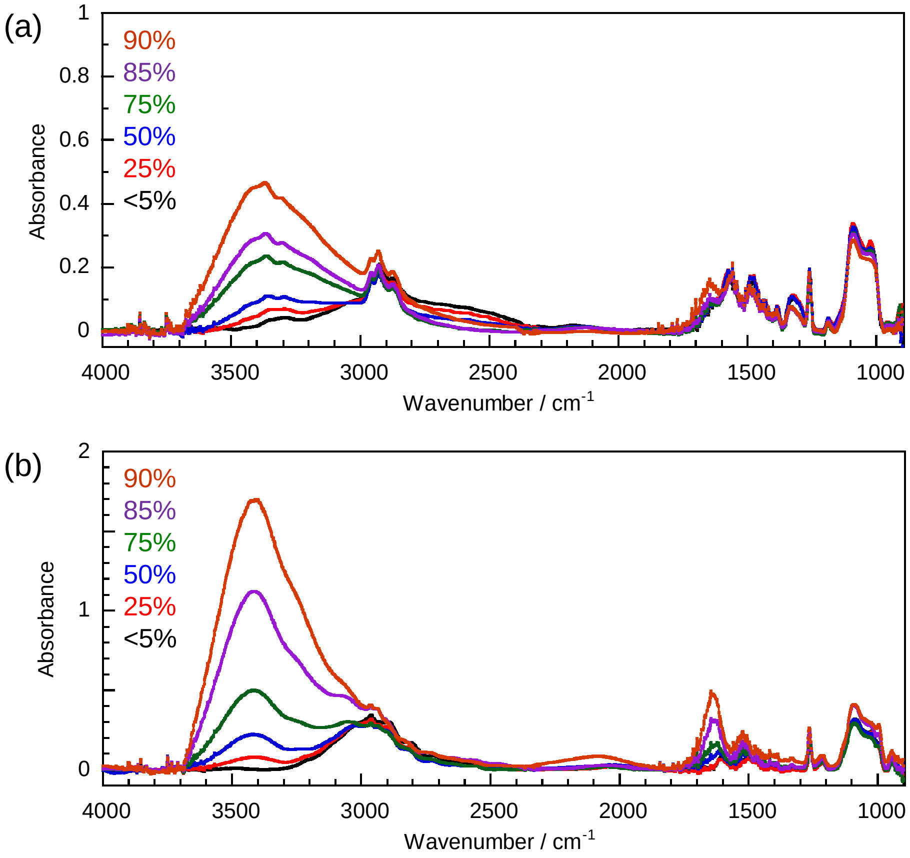

Fig. S4 Humidity-controlled IR spectra of (a) PSx(NH_2) and (b) PSx($NH_3^+Cl^-$).

Reversibility of moduli of the polysiloxane film

For the moist PSx($NH_3^+Cl^-$) spincast films, force curve measurements were performed. Before measurements, the spincast films were placed in the chamber with RH=60% overnight. The moist film was measured in an open system. After that, the film was exposed to the environment of N_2 atmosphere and force curve was recorded. Finally, we again measured the moist film exposed to RH=60% overnight. As shown in Fig. S5, the elastic modulus changed with the relative humidity. For the moist films, the elastic moduli could not be determined accurately because the humidity is not accurately controlled during measurements and the cantilever did not follow the retract process due to the plastic deformation of the film, but the repetitive humidity response of the modulus change could be qualitatively confirmed.

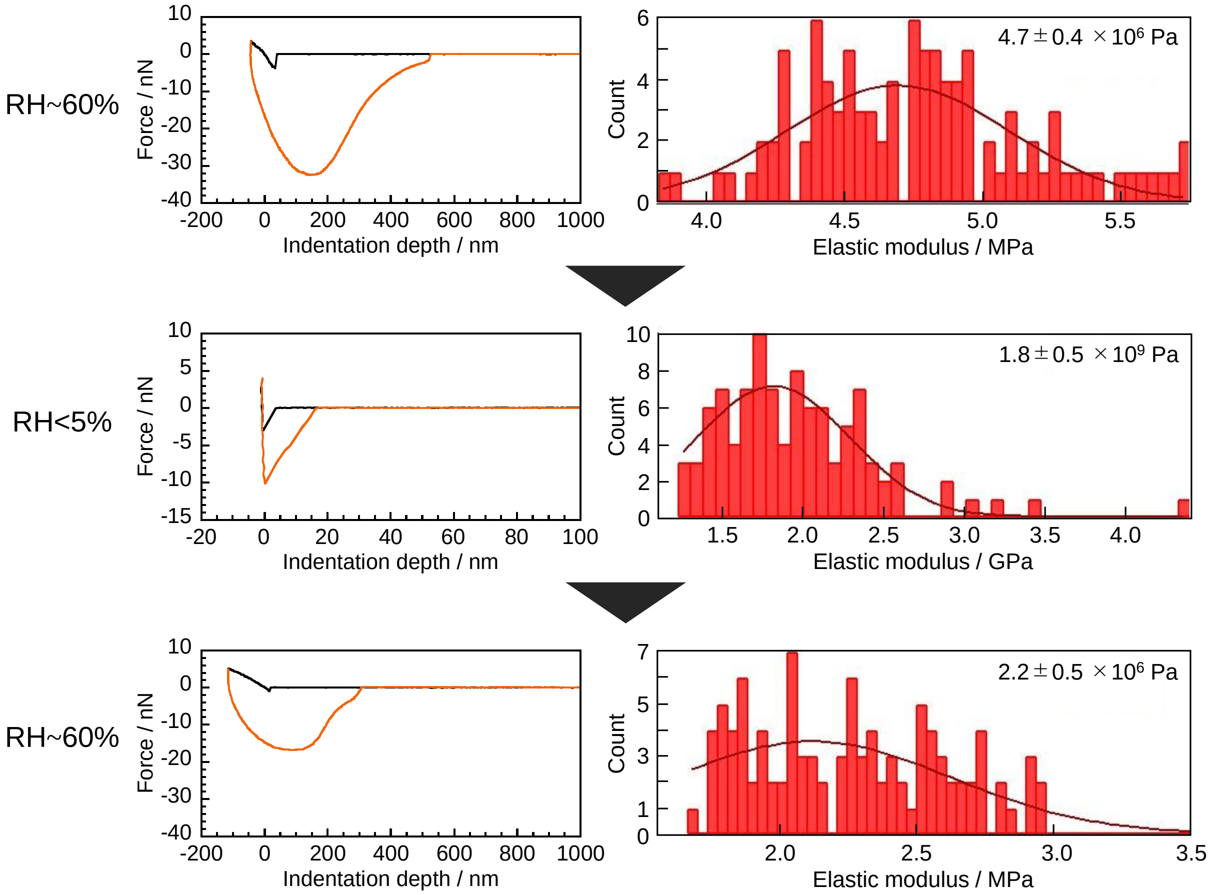

Fig. S5 Humidity-depending force curves and its elastic modulus histogram at 100 points.

XRD profile of the polysiloxane

As shown in Fig. S6a, PSx(NH_2) had no peak, indicating of amorphous structure. The amine group in PSx(NH_2) was hydrochlorinated by adding three times the equivalent amount of HCl to PSx(NH_2) and stirring at r.t. for 2h. The resulting PSx($NH_3^+Cl^-$) showed scatterings derived from lamellar structure (Fig. S6b). It means amine hydrochloride is necessary for the formation of the lamellar structure. The scatterings disappeared at RH=80%, suggesting the melting of the lamellar structure due to waster absorption (Fig. S6c). The scattering peaks appeared again upon dehumidification, and the formation and deformation of the lamellar structure occurred reversibly depending on the humidity.

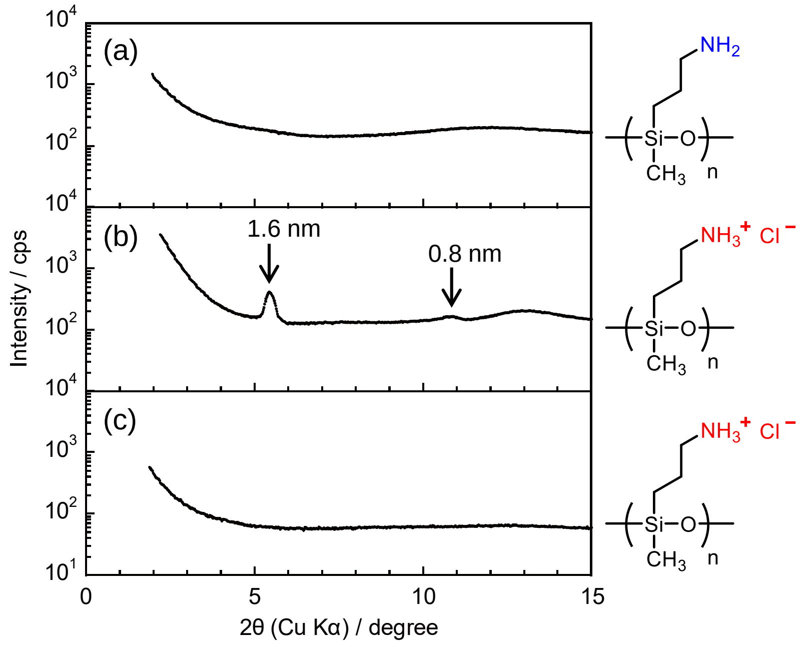

Fig. S6 XRD profiles of (a) PSx(NH_2) at less than 5% of relative humidity, (b) PSx($NH_3^+Cl^-$) at RH=25%, and (c) PSx($NH_3^+Cl^-$) at RH=80%. Temperature was set at 25 °C.

Hygroscopicity of polyallylamine hydrochloride

Polyallylamine hydrochloride (PAH, M_w: 1.75×10^4) was purchased from Sigma-Aldrich and evaluated for moisture absorption according to the procedure in the main manuscript. In Fig. S7, weight change ratios of ca. 50 mg of PAH and PSx($NH_3^+Cl^-$) depending on the relative humidity at 25 °C were shown. The moisture absorption behavior of PAH agreed well with that of PSx($NH_3^+Cl^-$), indicating that the amount of moisture absorption is determined by the hygroscopic group.

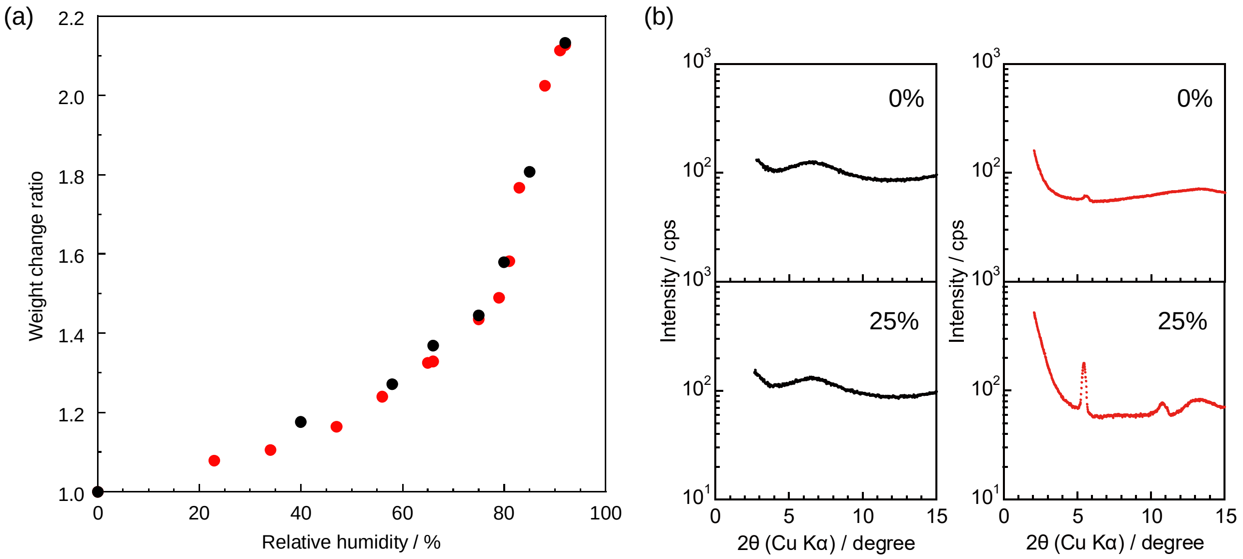

Fig. S7 (a) Weight change ratio and (b) XRD profiles of PAH (black) and PSx($NH_3^+Cl^-$) (red).

Test piece for shear adhesive force measurements

As shown in Fig. S8, test piece was assembled from two glass slides.

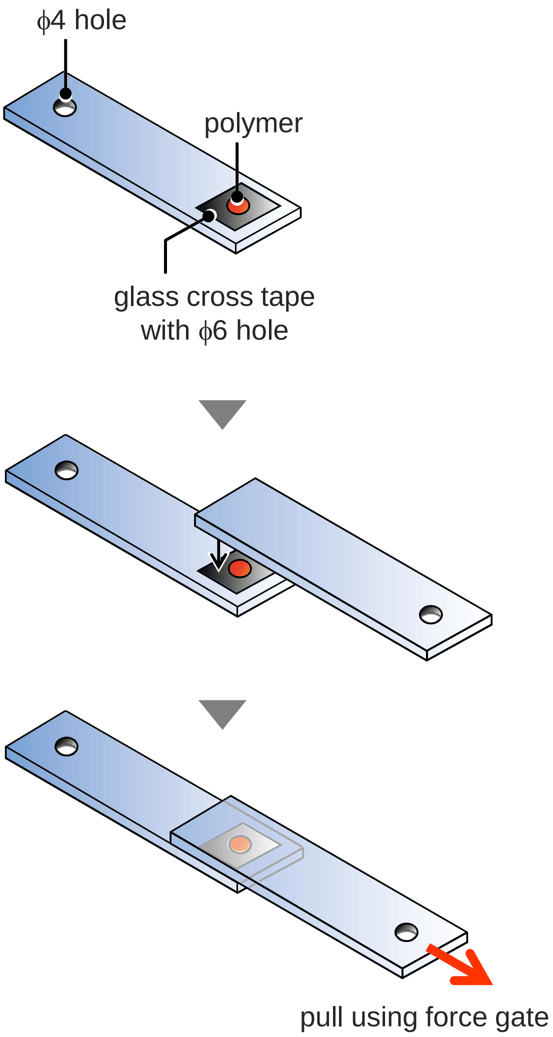

Fig. S8 Assembling process of test piece for shear adhesive force measurements.

Thermal stability

As shown in Fig. S9, a weight loss of 15% was observed at 100 °C, corresponding to the evaporation of water adsorbed on PSx($NH_3^+Cl^-$). No significant decrease was observed at higher temperature up to 260 °C. This means that PSx($NH_3^+Cl^-$) did not be pyrolyzed by heating up to o 260 °C.

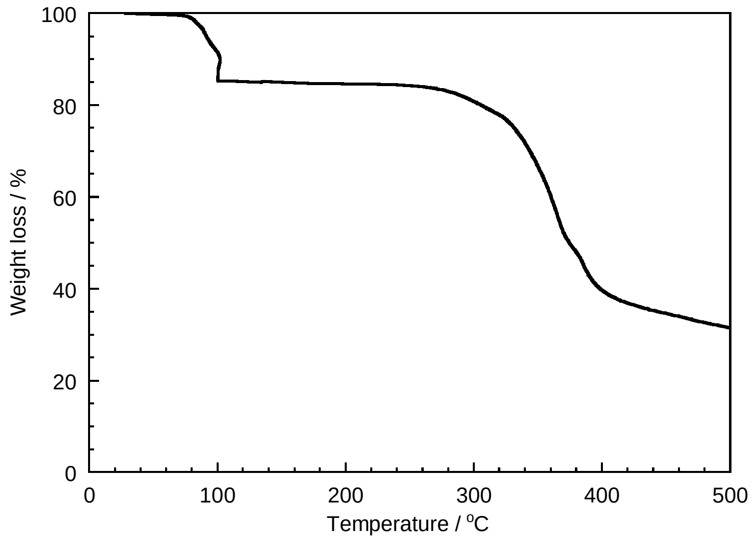

Fig. S9 TGA curve of PSx($NH_3^+Cl^-$).

Supplementary Movie 1

Adhesion test at RH=80%.

Supplementary Movie 2

Adhesion test immediately after drying at 100 °C under vacuum for 3 h.

References

1. Umemura, J., Kawai, T., Takenaka, T., Kodama, M., Ogawa, Y. & Seki, S. Fourier transform infrared study on the phase transitions of a water−dioctadecyldimethylammonium chloride system. *Mol. Cryst. Liq. Crtst.* **112**, 293−309 (1984).

2. Jentys, A.. Warecka, G., Derewinski, M. & Lercher, J. A. Adsorption of water on ZSM5 zeolites. *J. Phys. Chem.* **93**, 4837−4843 (1989).
